# Ontogenetic Changes in Auxin Biosynthesis and Distribution Determine the Organogenic Activity of the Shoot Apical Meristem in *pin1* Mutants

**DOI:** 10.3390/ijms20010180

**Published:** 2019-01-06

**Authors:** Alicja Banasiak, Magdalena Biedroń, Alicja Dolzblasz, Mateusz Adam Berezowski

**Affiliations:** Department of Plant Developmental Biology, Institute of Experimental Biology, Faculty of Biological Sciences, University of Wroclaw, Kanonia 6/8, 50-328 Wroclaw, Poland; magdalena.biedron@uwr.edu.pl (M.B.); alicja.dolzblasz@uwr.edu.pl (A.D.); mateusz.berezowski@uwr.edu.pl (M.A.B.)

**Keywords:** *Arabidopsis*, auxin immunolocalization, organogenesis, PAT, *pin1* mutant, *YUC* genes, SAM, vascular system, xylem

## Abstract

In the shoot apical meristem (SAM) of *Arabidopsis*, PIN1-dependent polar auxin transport (PAT) regulates two crucial developmental processes: organogenesis and vascular system formation. However, the knockout mutation in the *PIN1* gene does not fully inhibit these two processes. Therefore, we investigated a potential source of auxin for organogenesis and vascularization during inflorescence stem development. We analyzed auxin distribution in wild-type (WT) and *pin1* mutant plants using a refined protocol of auxin immunolocalization; auxin activity, with the response reporter *pDR5:GFP*; and expression of auxin biosynthesis genes *YUC1* and *YUC4*. Our results revealed that regardless of the functionality of PIN1-mediated PAT, auxin is present in the SAM and vascular strands. In WT plants, auxin always accumulates in all cells of the SAM, whereas in *pin1* mutants, its localization within the SAM changes ontogenetically and is related to changes in the structure of the vascular system, organogenic activity of SAM, and expression levels of *YUC1* and *YUC4* genes. Our findings indicate that the presence of auxin in the meristem of *pin1* mutants is an outcome of at least two PIN1-independent mechanisms: acropetal auxin transport from differentiated tissues with the use of vascular strands and auxin biosynthesis within the SAM.

## 1. Introduction

Organogenesis and differentiation of continuous vascular strands, linking newly developing organs with existing vasculature, are fundamental processes in plant development. These processes are related to the shoot apical meristem (SAM) and regulated by auxin [1,2]. In addition, both processes are interrelated and occur in the peripheral zone of the SAM [3], which surrounds the apical central zone composed of initial cells and the rib meristem, which is below the central zone and gives rise to the internal tissues of the stem [4,5,6,7]. In *Arabidopsis thaliana*, the SAM shows three distinct layers derived from the region where initial cells are confined. The most external L1 and underlying L2 layers, mostly dividing anticlinally, form the tunica, and internal L3 layer, with cells dividing in various planes, form the corpus of the SAM [5,6,7]. Organogenesis and vascular meristem (procambium) development are associated with cells of the L2 and L3 layers of the peripheral zone of the SAM, respectively [8,9].

Regulation of organogenesis and vascular differentiation is driven by transmembrane polar auxin transport (PAT). This type of auxin transport functions due to the activity of three groups of membrane transporters [10,11]: AUX1/LIKE-AUX1 (AUX1/LAX), facilitating the influx of auxin to the cell [12]; PIN-FORMED (PIN), mediating the efflux of auxin from the cell [1,13,14]; and ATP-BINDING CASSETTE SUBFAMILY B (ABCB) [15], also enabling the efflux of auxin out of the cell and probably regulating the level of auxin accessible for PIN proteins [10,16,17]. The direction of auxin flow, and thus auxin general distribution in the plant, depends mostly on the polar localization of PIN proteins in the plasma membrane [18], which can change in response to external and internal stimuli [19,20]. In *Arabidopsis*, eight PIN proteins are present, of which at least five (PIN1-PIN4 and PIN7) are localized in the plasma membrane, and are thus responsible for PAT [14,21]. However, their exact localization and relationship with auxin distribution are tissue and organ specific [22,23]. Interestingly, PAT within the SAM has been shown to be regulated solely by the PIN1 protein [6,24,25].

According to the currently accepted model, auxin is transported to the peripheral zone of the SAM acropetally in the epidermis and L1 layer using PAT and PIN1 proteins [1]. In this region, due to changes in PIN1 protein polarization, the maxima of auxin concentration are established, inducing organ primordia formation [1,20,26,27]. Simultaneous auxin depletion from the surrounding region generates the inhibitory field and the organ development in the specific spatial patterns. However, one has to bear in mind, that besides auxin, cytokinin is also involved in the establishment of organ distribution pattern [28,29]. Moreover, both hormones seem to interact in that process, as for example AHP6 (ARABIDOPSIS HISTIDINE PHOSPHOTRANSFER PROTEIN 6), an inhibitor of cytokinin signaling, involved in the establishment of the secondary inhibitory field outside of the developing primordium, is activated in response to auxin. In turn, cytokinin probably regulates the auxin concentration or the auxin response during new organ formation [28]. Auxin regulates also the subsequent development of the initiated organ primordia. First, it is transported using PIN1 proteins to their most apical part and then basipetally, within the internal layers and towards already existing vasculature, thus specifying the new vascular strand [1,20,30,31]. Consequently, the arising vascular strands join each other in the stem, establishing regular spatial patterns of the vascular strands in accordance with organ distribution in the stem. Analyzes of tomato and *Arabidopsis* shoots showed that the mechanism of organogenesis regulation and vascularization of the stem, driven by polar auxin transport, is similar in both vegetative and generative stages of plant development [1,32]. However, several studies have shown that besides PAT, signals from internal tissues are involved in the regulation of these two processes, although their role has not been deciphered yet [25,33,34,35,36].

Developing organ primordia start to biosynthesize auxin, which can be transported acropetally to the organogenic zone of the SAM, thus becoming a source of auxin for new organ primordia formation and differentiation of vasculature [27,37]. Some studies have indicated that auxin can also be synthesized directly in the organogenic (peripheral) zone of the meristem, which could be a source of auxin in addition to PAT [38,39]. Auxin biosynthesis in *Arabidopsis* mainly occurs by means of the tryptophan-dependent pathway, which is a two-stage process [40,41]. First, tryptophan (TRP) is converted to indole-3-pyruvic acid (IPA) due to the activity of one of the aminotransferases from the TRP AMINOTRANSFERASE of ARABIDOPSIS (TAA) family [42]. Next, IPA is transformed to indole-3-acetic acid (IAA) by flavin monooxygenases encoded by genes from the *YUCCA* (*YUC*) family [43]. In *Arabidopsis,* the aminotransferase gene *TAA1* and its two close homologues, *TAR1* and *TAR2*, were characterized and probably have a similar function [42,43]. Additionally, 11 *YUC* genes, with tissue-, organ-, and developmental stage-specific functions, were identified [39,44]. Their expression patterns, along with the phenotypes of their mutants, indicate that *TAA1*, *YUC1*, and *YUC4* genes are most important for auxin biosynthesis, regulating organogenesis on the SAM [39,42,44].

Auxin transport using PIN1 proteins is obligatory for organogenesis and vascularization in *Arabidopsis* [1,25,45,46,47,48]. Thus, mutation in the *PIN1* gene should inhibit both these processes. However, the generative stem of the *pin1* mutant is completely depleted of organs at early developmental stages, while single and malformed organs are produced at later stages [34,49,50]. Furthermore, the development of the vascular system is not blocked and only some patterning abnormalities and delays in the xylem differentiation are observed [34,45,50]. Thus, the main goal of our project was to analyze if, in *pin1* mutants, the organogenesis and vascularization are regulated, as in wild-type (WT) plants, by high auxin concentration. In addition, we investigated auxin potential sources when PIN1-dependent PAT is not functional. Our study showed that (1) in *pin1* mutants, organogenesis and vascularization are induced by high concentrations of auxin, established by means of synthesis in the meristem and acropetal auxin transport in vascular strands from differentiated tissues; (2) the source of auxin and its distribution within the meristem change during ontogenesis; (3) the meristem is active organogenically only when auxin is present in its superficial layers.

## 2. Results

### 2.1. Organogenic Activity of pin1 Mutant Inflorescence SAM

In order to examine if the organogenic activity of the meristem changes during the ontogenesis of the *pin1* generative stem, we analyzed the inflorescence stem morphology at different developmental stages. 

**Figure 1:**
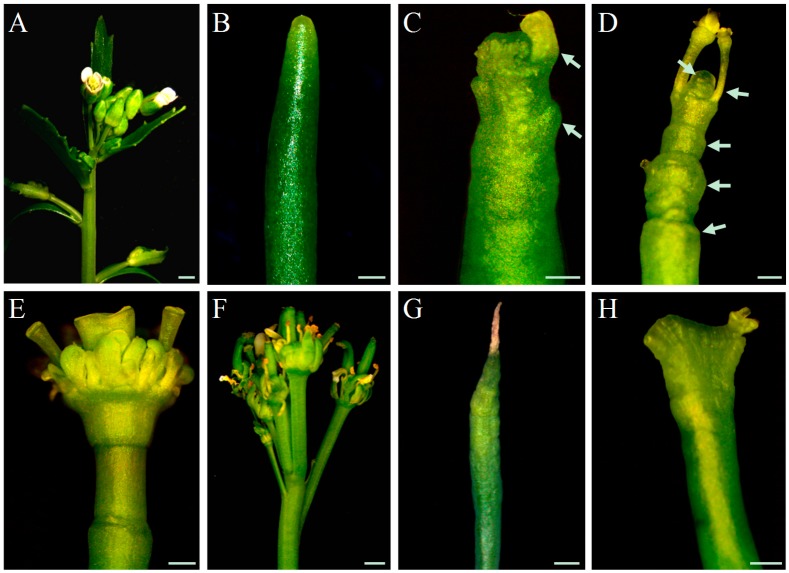
The phenotype of inflorescence shoots. The phenotype of the stem apex of wild-type (WT) (**A**) and *pin1* mutant (**B**–**H**) *Arabidopsis* plants. *pin1* mutant stems showing several developmental features of its apex: without organs (**B**); with single bulges and developing organ primordia (**C**), denoted with arrows; with numerous bulges, folds and malformed organs (**D**), denoted with arrows; a termination with a single organ (**E**), multiple organs (**F**), and meristem necrosis (**G**); fasciation (**H**). Scale bar 200 μm.

In WT plants, inflorescence stems are morphologically similar and flower primordia are densely packed on the meristem throughout all stages of inflorescence development (Figure 1A), until growth termination at the height of around 30 cm. In contrast, inflorescence stems of *pin1* mutants were phenotypically variable (Figure 1, Supplementary Table S2). However, some regularities related to the developmental stage were noticed. Young stems below 1 cm high were smooth, needle-like, and completely depleted of organs (Figure 1B), in higher stems (3–6 cm), single bulges and sporadically developing organ primordia were visible in the meristem (Figure 1C), and in older shoots of 10–15 cm height, the meristem led to the formation of multiple bulges, folds, and deformed organs, also visible in the mature part of the stem (Figure 1D). Growth of *pin1* mutants terminated when inflorescence stems were above 15–20 cm high and two phenotypes of stem growth termination were distinguished: (1) with the organogenic activity either in the form of a single flower-like structure with multiple elements (Figure 1E) or in the form of multiple deformed organs (Figure 1F); and (2) due to meristem necrosis (Figure 1G). These analyses showed that in *pin1* mutants, despite the damage of functional PAT, SAM can produce organ primordia and its organogenic activity gradually increases during the inflorescence stem development.

Based on the phenotypic changes described above for *pin1* mutants, we established five developmental stages for further analyses: stage I, stem height below 1 cm with no organogenic activity; stage II, stem height of 3–6 cm with sporadic organogenesis; stage III, stem height of 10–15 cm with intensive organogenesis; stage IV, stem height of 15–20 cm terminating with the formation of malformed organs; and stage V, stem height of 15–20 cm terminating with meristem necrosis. In addition, we observed fasciation of *pin1* mutant shoots (Figure 1H), but this phenotype was not analyzed in our study.

### 2.2. Structure of the Vascular System in Fully Developed Inflorescence Stems

Concomitantly, we analyzed how mutation in the *PIN1* gene, resulting in the damage of PAT in the SAM, and the occurrence of spontaneous organogenesis influence vascularization of the stem. We compared the spatial pattern of the vascular tissues arrangement in fully developed inflorescence stems of WT plants and *pin1* mutants on the series of successively prepared transverse sections from the base to the tip of the stem.

**Figure 2:**
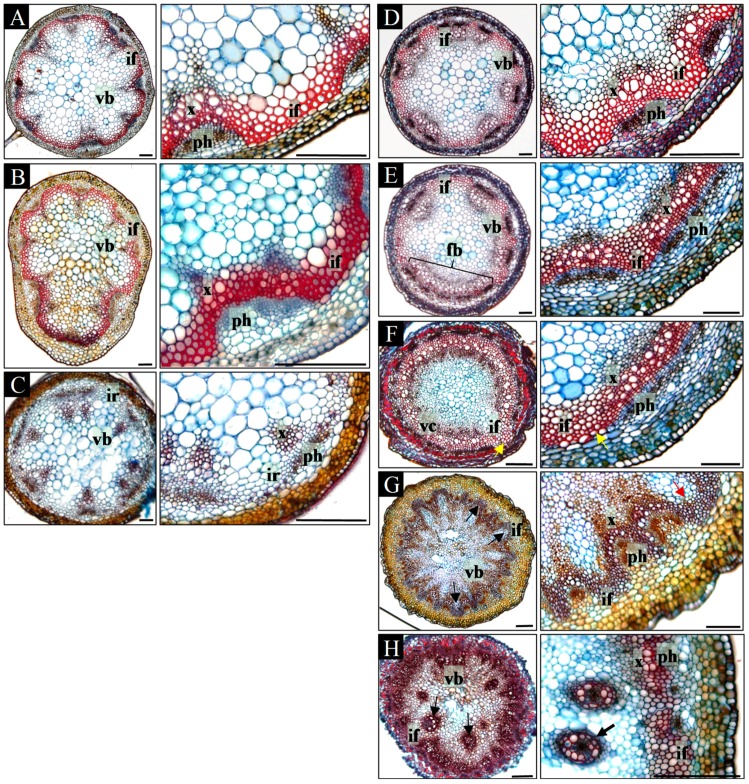
Ontogenetic changes in the structure of the inflorescence shoots. Subsequent cross-sections of the inflorescence shoot of WT (**A**–**C**) and *pin1* mutant (**D**–**H**) plants, showed in the basal-apical sequence. The left picture presents the vascular strands arrangement, and the right picture the magnification of the interfascicular region. The stem of the WT plant has in its basal part discrete vascular bundles (**A**); under the first branch increased number of bundles (**B**); and in the apical region the stem lacks differentiated interfascicular fibers (**C**). The stem of the *pin1* mutant is characterized by (looking from the most basal part of the stem towards the top) regions with: discrete vascular bundles (**D**); fusing bundles (**E**), denoted with bracket; the vascular cylinder, with interfascicular fibers displaced towards the pith (**F**), direction of fibers displacement denoted with arrow; vascular cylinder splitting into numerous small bundles, with interfascicular fibers displaced towards the stem surface (**G**), direction of fibers displacement denoted with arrows; concentric bundles in the pith of the stem (**H**), denoted with arrows. vb—vascular bundle, fv—fusing vascular bundles, vc—vascular cylinder, if—interfascicular fibers, ir—interfascicular region, x—xylem, ph—phloem. Scale bar 100 μm.

In the basal part of the mature inflorescence stems of WT plants, a regular pattern of 5–8 discrete vascular bundles was formed, between which interfascicular fibers were present (Figure 2A). A similar arrangement of vascular tissues was present at all positions within the acropetally analyzed inflorescence stems (Figure 2A–C). Beneath the lateral organs (leaves, branches, and flowers), the number of vascular bundles increased due to the formation of vascular traces extending into the lateral organs (Figure 2B). In addition, interfascicular fibers in the apical part of the stem were not differentiated (Figure 2C). Furthermore, in the basal part of 29% stems (*n* = 7), secondary growth was detected (Supplementary Figure S1A–D).

In contrast to the regular arrangement of the vascular tissues in WT plants, the vascular pattern in *pin1* mutants differed depending on the analyzed position within the inflorescence stem (Figure 2D–H). In the most basal part, the vascular system was composed of 7–10 discrete vascular bundles arranged similarly to WT plants (Figure 2D). Between xylem and phloem of neighboring bundles, interfascicular fibers were differentiated (Figure 2D). Additionally, in 61% (*n* = 23) mutant plants, secondary growth was observed, which varied circumferentially or was confined only to particular sectors (Supplementary Figure S1E–J). The arrangement of vascular bundles changed towards the tip of the stem. First, bundles fused laterally, and thus decreased in number (Figure 2E) until the continuous vascular cylinder was formed (Figure 2F). Only in one out of 23 stems it was fully continuous, as mostly 1–3 breaks were distinguished (Figure 2F). In addition, multiple interfascicular fibers pushed towards the pith were detected in the whole area between the xylem of neighboring bundles (Figure 2F). In a more acropetal direction, the vascular cylinder split into 7–10 bundles of different sizes, and then fused again forming a cylinder. Depending on the inflorescence stem, 1–4 split-fusion cycles could be detected and, interestingly, corresponded with the formation of the bulges and folds visible on the stem surface. Such split-fusion cycling ended up with the formation of a large number (13–18) of relatively small bundles of equal size (Figure 2G). In addition, the interfascicular fibers were shifted towards the surface of the stem and were observed between the phloem of neighboring bundles (Figure 2G). Further towards the tip of the stem, there were disturbances in the typical mutual arrangement of phloem, xylem, and interfascicular fibers (Figure 2H; Supplementary Figure S2). Consequently, locally interfascicular fibers surrounded the phloem (Supplementary Figure S2A,B) and numerous surplus concentric bundles were formed in the pith (Figure 2H; Supplementary Figure S2C,D). Such vascular tissue patterning was related to strong folding of the stem surface and the presence of malformed organs. In inflorescences that terminated their growth with meristem necrosis (stage V, *n* = 6) such an arrangement of vascular tissues was the last observed. In inflorescences terminating with organ formation, the vascular system split, supplying the particular organs (stage IV, *n* = 17).

Our analyses showed that in *pin1* mutants, the vascular system structure changes during ontogenesis, corresponding with the intensification of the organogenic activity of the SAM. Importantly, the damage of PAT, caused by mutation in the *PIN1* gene, does not stop vascular differentiation and also results in an increased number of formed vascular strands (vascular hypertrophy).

### 2.3. Ontogenetic Changes in the Spatial Arrangement of Xylem Strands in Inflorescence Stems

To better understand the relationship between organogenesis and vascularization in *pin1* mutants, we analyzed the spatial arrangement of xylem strands of inflorescence stems in the first three (described above) developmental stages (I–III). The height of the inflorescence stems of WT plants, examined as controls, corresponded to the height of the mutant stems in the same stage (depicted here as stages I–III of WT plants).

**Figure 3:**
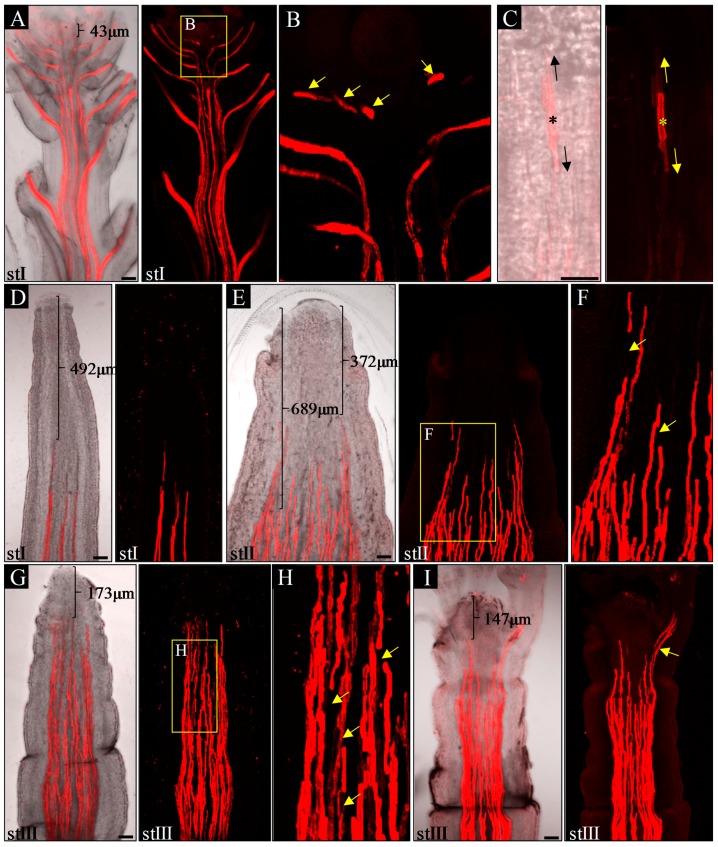
Structure of the xylem strands in the apices of the inflorescence stems, stained with propidium iodide. In WT plants, xylem strands differentiate close to the meristem—distance denoted with bracket, and are continuous in the mature stem and organs (**A**); discontinuities (denoted with arrows) are present only in the apical, meristematic region of the shoot (**B**). The discontinuities in the xylem strands, however, disappear during development, as a result of bidirectional xylem differentiation—denoted with arrows (**C**). *pin1* mutants are characterized by: discrete, continuous xylem strands differentiating far from the meristem (distance denoted with brackets), in stage I (**D**); increased number of xylem strands, in comparison to stage I, with variable distance from the meristem—denoted with brackets (**E**) and with local discontinuities—denoted with arrows (**F**), in stage II; numerous differentiated xylem strands close to the meristem—distance denoted with bracket (**G**), with frequent discontinuities—denoted with arrows (**H**), and developing malformed organs with their own xylem strands connected to the strands of the main stem—denoted by arrow (**I**), in stage III. St I–III—successive developmental stages, the asterisk—first differentiated protoxylem element. Scale bar 50 μm.

In inflorescence stems of WT plants, in all three analyzed stages (I–III), xylem vessels formed multiple, separate, and continuous strands connected to differentiated xylem strands of lateral organs (Figure 3A,B). The youngest detected protoxylem elements, differentiating in relation to organ primordia development, were observed below the SAM at an average distance of 93.3 μm from it (*n* = 10; Figure 3A, Supplementary Table S3). These elements were discontinuous with the protoxylem strands of the stem below (Figure 3B), a feature that was never observed in older organs due to the later bidirectional xylem strands differentiation (Figure 3C). Interestingly, in *pin1* mutants, the spatial structure of xylem strands changed ontogenetically. In young pin-like inflorescence stems (stage I), without any signs of organogenesis, protoxylem vessels formed discrete strands, which were at an average distance of 602 μm from the meristem (*n* = 7; Figure 3D, Supplementary Table S3), further than in WT plants. These strands were always continuous, suggesting their unidirectional acropetal differentiation. In the stems of stage II plants, with single bulges or folds, xylem strands were less regular in their axial orientation and were observed less distant at an average distance of 373.7 μm (Figure 3E, Supplementary Table S3). These strands were often connected with each other in an unpredictable manner (Figure 3E) and were characterized by sporadic discontinuities (Figure 3F). Furthermore, within one inflorescence stem, particular strands differed in their distance from the meristem, with protoxylem located closer to the meristem in sectors with emerging organ primordia (Figure 3E). In stage III, with strong stem folding and presence of malformed organs, the number of discontinuities of xylem strands increased (Figure 3G,H) and the youngest detected protoxylem elements differentiated very close to the meristem (average distance of 171 μm; Figure 3G, Supplementary Table S3). Malformed organs were characterized by their own differentiated protoxylem, continuous with the vascular system of the stem (Figure 3I), while stem folds and bulges lacked their own vascular system.

These analyses revealed that ontogenetic changes in the structure of the vascular system in *pin1* mutants, such as non-simultaneous maturation of the xylem strands, discontinuities in the vascular system, and vascular hypertrophy, are closely related to the increase in organogenic activity of the SAM.

### 2.4. Auxin Distribution during Inflorescence Stem Ontogenesis

In order to analyze how auxin distribution at the tissue level changes during inflorescence stem ontogenesis in *pin1* mutants, we took advantage of transgenic lines with synthetic auxin response promoter DR5 (WT; p*DR5:GFP* and *pin1*; p*DR5:GFP*) and fine-tuned the protocol for auxin immunolocalization.

**Figure 4:**
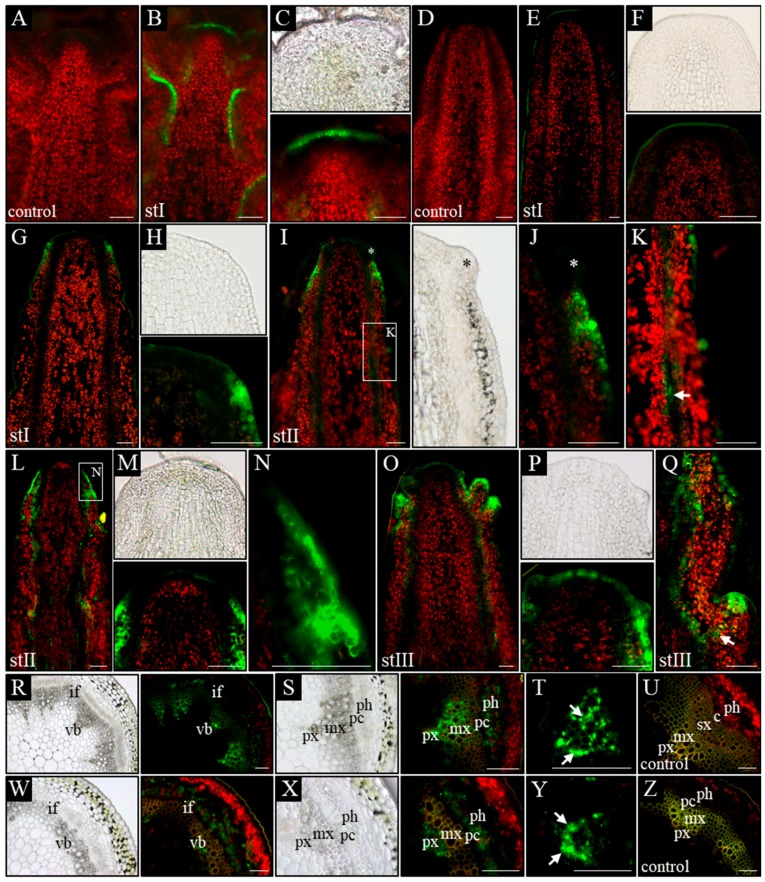
Spatial localization of the auxin response reporter p*DR5:GFP* activity. Longitudinal (**A**–**Q**) and transversal (**R**–**Z**) sections through the apical part of inflorescence stems of WT (**A**–**C**,**R**–**U**) and *pin1* mutant (**D**–**Q**,**W**–**Z**) plants. On the longitudinal sections, in WT plants, the GFP signal is visible in the vascular strands (**B**) and the L1 layer of the meristem (**C**). In the *pin1* mutant, in stage I, the activity of the p*DR5:GFP* transgene is not detected (**E**,**F**) or is present in the L1–L2 layers of the peripheral zone of the meristem (**G**,**H**); in stage II, GFP signal accumulates in the surface layers at the base of the organ primordia—primordium denoted with asterisk (**I**,**J**) and locally in the vascular bundles - denoted with arrow (**K**) or in the L1–L2 layers of the peripheral zone of the SAM and in all vascular bundles (**L**–**N**); in stage III, the GFP signal is visible in the L1 layer of the entire meristem, at the tip of the developing organs and in all vascular strands (**O**,**P**) as well as of the cells (denoted with arrow) connecting the tip of an organ primordia with the vasculature (**Q**). On the transversal sections, the activity of the p*DR5:GFP* transgene in WT plants is visible in the phloem, xylem and locally in procambial cells (**R**–**U**). In terms of the xylem, GFP signal accumulates in all xylem parenchyma cells—denoted with arrows (**S**,**T**). In the *pin1* mutant GFP signal is visible in xylem and phloem (**W**–**Z**). In terms of the xylem, GFP signal is present only in the parenchyma cells on the protoxylem side—denoted with arrows (**Y**). (**A**,**D**,**U**,**Z**) Tissue autofluorescence (control); (**C**,**F**,**H**,**J**,**M**,**P**) magnifications of the meristems. St I–III—successive developmental stages; vb—vascular bundle, if—interfascicular fibers, px—protoxylem, mx—metaxylem, sx—secondary xylem, ph—phloem, pc-procambium, c—cambium. Scale bare 50 μm.

In control plants (WT), in all analyzed developmental stages (I–III), a signal from the p*DR5:GFP* was present in the L1 layer of the meristem, surface layer of organ primordia, and all vascular strands (Figure 4B,C). Furthermore, in vascular strands from the differentiated region, GFP was detected in the xylem parenchyma cells, phloem, and locally in the procambium (Figure 4R–T). In *pin1* mutants, p*DR5:GFP* expression changed ontogenetically. In stage I, the GFP signal was usually not detected in the meristem and vascular strands, even when xylem elements were differentiating (Figure 4E,F). Interestingly, in one out of six stems of that stage, the signal was present in the outer layers (L1 and L2) of the lower part of the peripheral zone of the meristem, probably predating organogenic activity of the SAM (Figure 4G,H). In stems of stage II, with early-appearing small bulges on the meristem, the auxin response signal was detected at the base of these bulges (Figure 4I,J). The signal was stronger in the L1 and L2 layers, but weaker in more internal tissues and locally in vascular strands (Figure 4K). In this stage, if several bulges and/or malformed organs were present (Figure 4L–N), a strong expression of the p*DR5:GFP* was additionally detected in all vascular strands (Figure 4L). Interestingly, during this developmental stage, the GFP signal was not detected at the tip of the meristem, nor at the tip of developing organ primordia (Figure 4I–M). In stage III, the oldest analyzed mutant stems, p*DR5:GFP* expression in the meristem changed and was detected in the whole L1 layer of peripheral and central zones of the meristem (Figure 4O,P). Additionally, the p*DR5:GFP* signal was detected in the tips of initiated bulges, vascular strands of the stem, and the cells connecting these two regions (Figure 4Q). In the mature region of stage III stems, the p*DR5:GFP* expression localized to the xylem and phloem of vascular bundles, but was not observed in the undifferentiated cells of the procambium (Figure 4W–Z). In the xylem, the GFP signal was only detected in the parenchyma cells on the protoxylem side (Figure 4Y).

**Figure 5:**
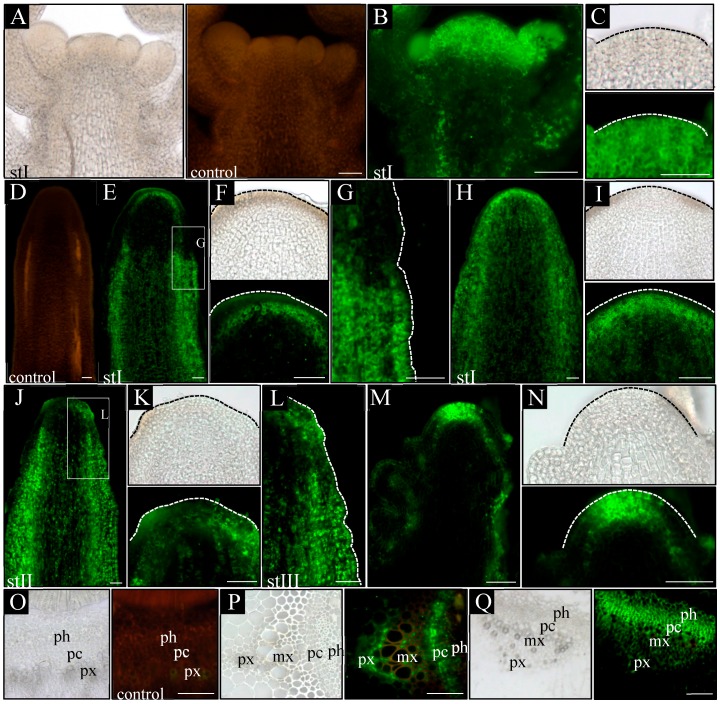
Immunolocalization of auxin in the inflorescence stems. Longitudinal (**A**–**N**) and transversal (**O**–**Q**) sections through the apical part of the inflorescence stems of WT (**A**–**C**,**O**–**P**) and *pin1* mutant (**D**–**N**,**Q**) plants. On the longitudinal sections, in WT plants, the strongest signal of auxin immunolocalization is visible in the entire meristem, developing organs and all vascular strands (**B**,**C**). In the *pin1* mutant, in stage I, the auxin signal is visible in the L3 layer of the meristem, vascular strands of the apical part of stem (**E**,**F**) and in all cell types of the more basal stem region (**G**), or in all cells of the entire shoot, except the surface layers (**H**,**I**); in stage II, the auxin immunolocalization signal is additionally visible in the L1 layer of the peripheral zone (**J**,**K**), and the more basal region, characterized by the presence of the auxin signal in all cells, is closer to the meristem (**L**); in stage III, the immunolocalization signal is visible in the L1–L3 layers of the entire meristem, the surface layer of the organs and all vascular strands (**M**,**N**). On the transversal sections, in WT plants, auxin immunolocalization signal accumulates in the procambial region, phloem and all cells of xylem parenchyma (**P**). In the *pin1* mutant, the signal is visible in the procambial region, phloem, and xylem parenchymal cells on the protoxylem side (**Q**). (**A**,**D**,**O**) Tissue autofluorescence (control); (**C**,**F**,**I**,**K**,**N**) magnifications of the meristems, meristem outlined with dashed line; (**G**,**L**) magnifications of the marked regions, the surface of stem outlined with dashed line. St I–III—successive developmental stages; px—protoxylem; mx—metaxylem; ph—phloem; pc—procambium. Scale bar 50 μm.

Next, we directly visualized auxin in the inflorescence stems at different developmental stages by a fine-tuned protocol of auxin immunolocalization. In WT plants, a similar pattern of auxin distribution was detected in all three analyzed stages (I–III). Auxin was present in all cells of shoot tips with clearly higher levels in the meristem, organ primordia, and developing vascular strands (Figure 5B,C). In the basal part of the analyzed stems, the strongest signal was detected in vascular strands, where auxin was confined to the procambium, phloem cells, and xylem parenchyma (Figure 5P). Auxin immunolocalization in *pin1* mutants revealed that in young needle-like stems, in developmental stage I, two different patterns of auxin distribution occurred. In the most common pattern, present in seven out of nine shoots, auxin distribution was different in the apical and basal part of the stem (Figure 5E–G). In the apical region, auxin was detected only in developing vascular stands and connected with them the internal layers of the meristem (Figure 5E,F), whereas in the basal part, the auxin signal was present in all tissues except the epidermis, where it was detected only locally (Figure 5E,G). The strongest signal was detected in vascular strands, where auxin was confined to the procambium, differentiating and differentiated phloem cells, and single cells of the xylem (Figure 5Q). Interestingly, there was a clear boundary between the basal and apical pattern of auxin distribution in the same shoot (Figure 5E–G). In the second pattern of auxin distribution in stage I, observed only in two out of nine analyzed stems, auxin was present in all cells of the stem, with the exception of the surface layers (Figure 5H,I). In addition, the immunolocalization signal was stronger in vascular strands and inner layers of the meristem.

In later stages of *pin1* mutants’ development, the auxin distribution pattern was always different between the apical and basal parts of the analyzed stem fragments. In stage II, in the apical part auxin was localized in the vascular strands, inner layers of the meristem, and locally in the L1 layer of the peripheral zone of the SAM (Figure 5J,K). The signal in the L1 layer was strongly associated with developing organ primordia, which sporadically started to appear on the meristem during this stage (Figure 5K). In the basal part, auxin was detected in all cell types of the inflorescence stem, similar to the most common pattern of auxin distribution in stage I (Figure 5J,L). However, this basal pattern of auxin distribution was detected closer towards the tip of the stem than in stage I (Figure 5J,L). In stage III, auxin distribution changed only in the apical region in comparison to stages I and II. In these stems, the strong auxin signal was present in all meristem layers, where normally initial cells are maintained, and in vascular strands connected to the L3 layer (Figure 5M,N).

The experiments using the auxin response marker p*DR5:GFP* and auxin immunolocalization showed that auxin distribution in the meristem of *pin1* mutants changes ontogenetically and the organogenic activity of the meristem is linked to the presence of auxin in the L1 layer. Additionally, the p*DR5:GFP* expression pattern in the meristem does not fully reflect the pattern of auxin distribution visualized with the use of immunolocalization, but is strongly related to organogenesis.

### 2.5. Ontogenetic Changes in Auxin Biosynthesis

To examine if auxin is synthetized in the inflorescence meristems of *pin1* mutants, we analyzed the expression pattern of *YUC1* and *YUC4* genes in different developmental stages using transgenic lines p*YUC1:GUS* and p*YUC4:GUS*, and quantitative reverse-transcription polymerase chain reaction (qRT-PCR).

**Figure 6:**
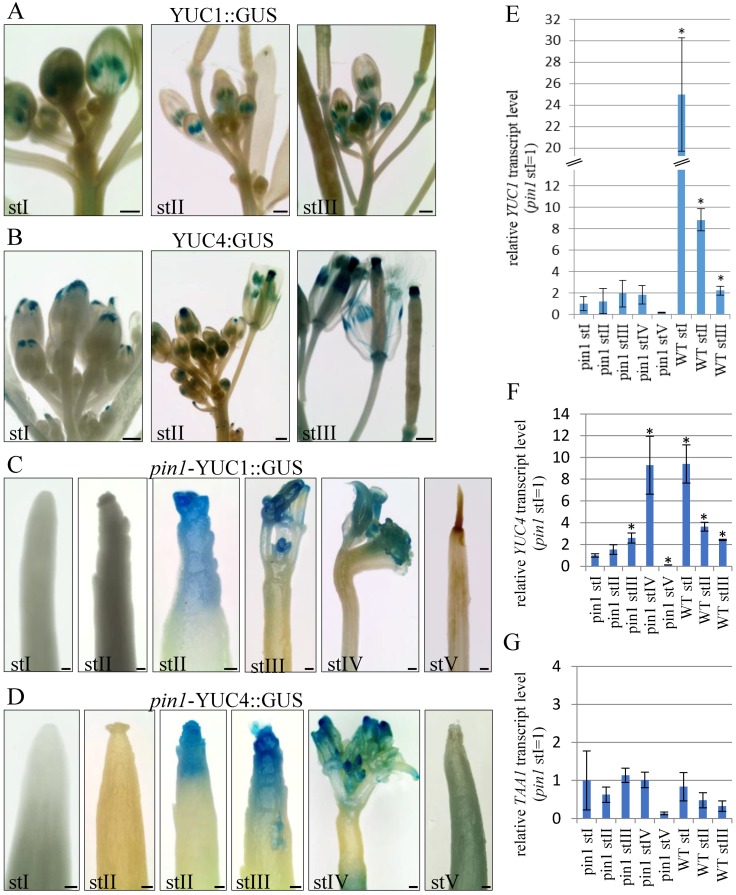
Expression analyses of auxin biosynthesis genes *YUC1* and *YUC4*. (**A**–**D**) Activity of the *YUC1* (**A**,**C**) and *YUC4* (**B**,**D**) promoters visualized by the activity of the GUS reporter protein (blue). In WT plants, in stages I-III, activity of the p*YUC1:GUS* (**A**) and p*YUC4:GUS* (**B**) reporters is visible in developing organs. In *pin1* mutants the expression of p*YUC1:GUS* (**C**) and p*YUC4:GUS* (**D**) is not detected in stage I plants; is visible in some shoots with organ primordia in stage II plants; is present in most plants from stages III and IV; and is not detected in stage V plants. (**E**–**G**) The comparison of *YUC1* (**E**), *YUC4* (**F**) and *TAA1* (**G**) transcript levels during different ontogenetic stages of inflorescence shoots development of WT and *pin1* mutant plants. Abundance of the transcripts, in each case, is relative to the *pin1* stage I sample. The level of *YUC1* and *YUC4* genes expression in *pin1* mutants increases during ontogenesis, but yet is generally weaker in comparison to WT plants. The level of *TAA1* gene expression in WT plants and *pin1* mutants does not generally significantly differ. Statistically significant differences to the *pin1* stage I sample are denoted with asterisk (*t*-test, significance at *p* = 0.05). St. I–V (*pin 1*) and St. I–III (WT)—successive developmental stages. Scale bar 500 μm.

In WT plants, p*YUC1:GUS* and p*YUC4:GUS* expression was detected during all three (I–III) analyzed stages of inflorescence stem development (Figure 6A,B). Both transgenes had strong expression in young inflorescence stems of stage I, when flower buds just started to develop (Figure 6A,B). *YUC1* promoter activity was visible at the flower receptacle, where flower elements are formed, and temporary in stamens (Figure 6A). *YUC4* promoter activity localized both in the flower receptacle and in the tips of petals (Figure 6B). In stems of stage II, with some flowers already fully developed, p*YUC1:GUS* was expressed in stamens and flower receptacles, where it could be detected even after silique formation (Figure 6A). p*YUC4:GUS* expression in this stage completely disappeared from the tips of petals of fully developed flowers and was detected in the flower receptacle, stamens, and the tips of carpels (Figure 6B). After seed production, in stage III, p*YUC4:GUS* expression was maintained in the apical and basal parts of siliques (Figure 6B). Thus, *YUC1* and *YUC4* promoter activity in WT plants clearly decreased with flower maturation and siliques formation. This was further confirmed by qRT-PCR analyses of *YUC1* and *YUC4* genes transcript levels (Figure 6E,F).

In *pin1* mutants, p*YUC1:GUS* and p*YUC4:GUS* expression in the inflorescence stems was similar and depended on the developmental stage (Figure 6C,D). In young shoots, in stage I, the activity of p*YUC1:GUS* (*n* = 12) and p*YUC4:GUS* (*n* = 12) was never detected in the meristem (Figure 6C,D). During stage II, GUS signals in the meristem were detected in 31.6% of plants for *YUC1* (*n* = 19; Figure 6C) and in 25.9% of plants for *YUC4* (*n* = 27; Figure 6D). Expression of none of these reporters was directly related to the organogenic activity of the meristem, because in 62.5% p*YUC1:GUS* (*n* = 16) and 69.6% p*YUC4:GUS* (*n* = 23) plants, the GUS signal was not detected in the shoot apex, despite the presence of the single bulges, folds, or developing organ primordia (Figure 6C,D). Importantly, in mutant plants where *YUC1* or *YUC4* promoter activity was detected, the GUS signal was weak and located in the region encompassing the meristem and/or tips of organ-like structures (Figure 6C,D). In stage III, *YUC1* and *YUC4* promoter activity was detected in 48% (*n* = 25) and 58.33% (*n* = 24) of analyzed plants, respectively (Figure 6C,D). When present, for both genes, the GUS signal was detected in the most apical part of the inflorescence stem of mutants (Figure 6C,D). In stems of stage IV terminating with organogenesis, *YUC1* promoter activity was detected in 45.45% plants (*n* = 11), where the signal localized to developing malformed organs (Figure 6C), and *YUC4* promoter activity was detected in developing and fully developed organs of 78.57% of the analyzed plants (*n* = 13; Figure 6D). In shoots terminating with meristem necrosis (stage V), neither *YUC1* (*n* = 9) nor *YUC4* (*n* = 11) promoter activity was detected in the stem apex (Figure 6C,D).

Next, we examined *YUC1* and *YUC4* expression using qRT-PCR and revealed that transcript levels of both genes in *pin1* mutants, in all five developmental stages (I–V), were much lower than those of WT plants (Figure 6E,F). In the case of the *YUC4* gene, its expression in *pin1* mutants significantly increased in stages III and IV compared to stage I. In stage V terminating with meristem necrosis, there was a marked decrease in the expression of the *YUC4* gene (Figure 6E,F). However, regarding the *YUC1* gene, there was no statistically significant differences in its expression levels between different developmental stages, though the tendency of changes was the same as that detected for the *YUC4* gene (Figure 6E,F). In addition, we analyzed the expression of *TAA1*, a gene coding for an enzymatic protein catalyzing earlier stages of auxin biosynthesis than the *YUC* genes, but still on the same pathway dependent on IPA [40,42]. Interestingly, *TAA1* transcript levels, in both WT and mutant plants, did not significantly differ ontogenetically and decreased only at the latest developmental stage (Figure 6G).

The performed analyses indicated that auxin is synthetized in the shoot apex of *pin1* mutants at later stages of inflorescence stem development, when meristems are characterized by increased organogenic activity.

## 3. Discussion

Postembryonic formation of the lateral organs on the SAM and development of the vascular system are two linked processes [2,31,51] in which a crucial role is played by auxin, which is transported in a polar manner due to the activity of PIN1 proteins [1,6,47]. Thus, a dependent-on-PAT model explaining auxin regulation of both processes was formulated [1,27,52]. However, it is evident that PAT alone does not fully explain how the position of a new organ and vascular strand continuous with the existing vasculature, are determined, and therefore the existing model should be extended [24,25,34,35]. We showed that in *pin1* mutants, organogenesis and vascular differentiation are regulated by auxin, which is supplied to SAM by additional, independent to PIN1-mediated PAT, mechanisms.

The lack of organ initiation in needle-like stems of *pin1* mutants has been previously associated with the damage of functional PAT as the source of auxin for organogenesis [1,27]. This was supported by the phenotypes of plants grown in the presence of the PAT inhibitor NPA [45] and the lack of expression of the synthetic reporter of auxin response p*DR5:GUS* in meristems of plants lacking functional PIN1-dependent PAT [25,37]. On the other hand, sporadic organogenesis indicated the presence of auxin in the SAM of those plants [34,49,50]. Furthermore, the results of the present study showed that organogenic activity of the SAM increases during ontogenesis and is accompanied by vascular hypertrophy, suggesting that auxin accumulates gradually. Therefore, to explain this, experiments with auxin immunolocalization were conducted. The present study for the first time showed the pattern of auxin distribution in the SAM of *Arabidopsis* plants in a direct way. The results showed that auxin is present in the meristem regardless of whether PIN1-dependent PAT is functional (WT plants) or not (*pin1* mutants), suggesting that PAT can be not the only source of auxin for the meristem.

Although auxin is present in the meristem, in *pin1* mutants, its localization changed ontogenetically. We identified three patterns of auxin distribution in the SAM depending on the developmental stage of the mutant: (1) present only in inner layers of the meristems, (2) present in inner layers and locally in surface layers of the peripheral zone, (3) present in inner and surface layers of the whole meristem. The first pattern was detected in young needle-like stems, with completely arrested organogenesis, suggesting that auxin in the inner layers (L3) is not sufficient to induce organ formation. Organogenesis was always related to the second and third patterns, when auxin was present also in surface layers (L1 and L2) of the meristem. Thus, our data are compatible with previous studies demonstrating the importance of the L1 layer and PIN1 protein expression in the outer layers, for the presence of auxin in the meristem and organogenesis [25,31,53]. However, we additionally showed that auxin-inducing organogenesis is present in the outer layers of the SAM in plants lacking functional PIN1 proteins, proving that auxin in the L1 layer, which induces organogenesis, can originate from other sources besides PIN1-mediated PAT. 

Our analyses showed that the meristem of *pin1* mutants is organogenically active only in later stages (II and III) of the inflorescence stem development. The increased organogenesis in stage III was associated with the increased expression of *YUC1* and *YUC4* genes, which are responsible for the final steps of auxin biosynthesis via the IPA pathway in the SAM [39,44]. Thus, it is highly plausible that the presence of auxin in outer layers of the meristem is the outcome of auxin synthesis, which becomes the source of this hormone for organogenesis in not-functional PIN1-dependent PAT conditions. Interestingly, the experiments of Cheng et al. [39] indicate that in WT plants, auxin biosynthesis can function together with PAT in organ initiation; however, the underlying mechanisms remain unclear [6]. Importantly, organogenesis in *pin1* mutants probably can occur also without auxin biosynthesis in the IPA pathway as, particularly in stage II of stem development, organs were initiated even without detectable expression of p*YUC1:GUS* and p*YUC4:GUS* in the SAM. This suggests that organogenesis-inducing auxin present in the L1 layer can also be transported from the outside of the meristem, although the possibility of auxin synthesis by a different pathway than IPA cannot be fully ruled out. Such a dual source of auxin, biosynthesis and transport, for organogenesis in case of PAT damage, is further suggested by the presence of two different patterns of auxin and auxin response distribution in organogenically active meristems of *pin1* mutants. 

The most probable routes of auxin transport to the SAM are the developing vascular strands, since, at each developmental stage, they are characterized by high auxin levels and are coupled to the L3 layer of the meristem, which always contains auxin. Such a transporting route was previously widely suggested [33,34,37,54,55,56]; nevertheless, it has never been experimentally proven. Previous studies on tissue extracts showed that inflorescence stems of *pin1* mutants have similar levels of auxin to WT plants, and thus it was proposed that auxin in mutant stems is transported acropetally through the phloem [56]. Our immunolocalization experiments directly showed high auxin concentration in the phloem and procambium cells of the vascular bundles of *pin1* mutants. At the tip of the stem, auxin presence was usually limited to these cells, and the activity of PAT independently of PIN1 (which could be responsible for the transport of auxin to the apoplast) was previously ruled out [24,25]. It is thus possible that in *pin1* stems that lack organs, auxin is transported to the L3 layer of the meristem acropetally through the cells of the procambium and protophloem using the symplasmic transport pathway.We cannot completely rule out the presence of auxin transport outside the protoplast with the participation of other transporters; however, auxin outside the phloem-procambium system in this stage was not detected in the SAM. Interestingly, symplasmic transport with use of plasmodesmata was recently shown to be involved in gravitropic reactions in plants with inhibited PAT [57,58].

There is still an open question regarding how auxin reaches the peripheral zone of the meristems at stage II of stem development. In stems with auxin detected in the peripheral zone, the basal region, in which auxin was detected in all cell types, was closer to the meristem. Interestingly, in these stems, differentiated elements of the protoxylem were also closer to the meristem. Because previous research on *pin1* mutants showed that organ initiation on the meristem is strictly correlated with the location of the youngest xylem elements in regard to the organogenic zone [34], it is plausible that these elements are the source of auxin and induce organogenesis in the peripheral zone. Moreover, auxin was detected in the xylem sap of *Ricinus* by Baker [59], and previous physiological studies on maize showed that auxin can be transported acropetally, probably in the xylem sap, as a conjugate [60]. Thus, it cannot be excluded that in *Arabidopsis*, auxin is transported in a similar way, then spreads via the apoplast, and afterwards is metabolized in the meristem, gaining biological activity. Importantly, the previously estimated [32] range of diffusion of auxin able to induce organogenesis is relatively similar to the distance between the youngest protoxylem elements and the meristem in stems, where auxin was detected in the L1 layer of the SAM. Another possible route for auxin transport to the organogenic zone is its unloading from the symplasmic pathway (via phloem and/or procambium cells) to the apoplast with the use of PIN proteins other than PIN1. Notable candidates are PIN3 and PIN7 proteins, which seem not to be present in the meristem [24,25], but were detected in mature stem regions with already differentiated tissues [61,62].

Numerous studies indicate that auxin transported, with the use of PIN1 proteins, in a polar manner from the organ tips in the direction of already existing vasculature, regulates all stages of vascular connections formation [45,47,63,64,65,66]. However, our results are in agreement with research showing that the presence of PIN1 in inner layers of the meristem is not necessary for the aforementioned process to occur [25]. Additionally, we showed that the vascular system develops and is capable of the formation of functional connections with lateral organs without the presence of functional PIN1 proteins, as in *pin1* mutants. Nonetheless, the presence of high auxin concentrations seems obligatory. As other PIN proteins were not detected in the meristem of *pin1* mutants [24,25], other mechanisms besides PAT can be responsible for the presence of auxin and the induction of differentiation of vascular strands. Such mechanisms could be auxin acropetal transport from the leaf rosette via vascular tissues and, in later developmental stages, induction of auxin biosynthesis via the IPA pathway. 

Older stems of the *pin1* mutant are characterized by numerous discontinuities within the xylem strands. The discontinuous fragments subsequently differentiate in the direction of other, already differentiated elements, concomitantly establishing a connection. This suggests that the differentiated vascular strands are the source of the signal guiding the differentiation. As auxin is present in the vascular tissues, it is plausible that it constitutes this signaling molecule. Discontinuities were also observed in the venation pattern of leaves in the *van3/sfc* mutant [67,68]. However, in this case, the formation of the connections did not follow during development. The *VAN3(VASCULAR NETWORK DEFECTIVE3)/SFC (SCARFACE)* gene is induced by auxin but its activity in leaf vascularization is independent of PAT [67]. Therefore, it is possible that the *VAN3* gene function is related to auxin transported in the vasculature, and probably is necessary to establish the continuous vascular strands.

In stems lacking organs, the vascular system forms differentiating acropetally discrete continuous strands in which, despite high auxin concentration, the expression of the synthetic promoter of auxin response (p*DR5:GFP*) was not detected. Moreover, in stems with organogenically active meristems, the differentiation of vascular strands occurs in parallel with the loss of its discrete character and the occurrence of multiple discontinuities. Additionally, differentiation of the vascular system in the presence of lateral organs was always associated with the clearly distinguishable expression of the auxin response reporter (p*DR5:GFP*), which was detected in developing organs and vascular strands. Vascular strand differentiation may therefore occur both dependently and independently to the presence of lateral organs on the meristem, probably employing two different mechanisms. One mechanism is unrelated to organogenesis and not activating the auxin response reporter, and the other is always associated with organogenesis and activating the reporter response. Therefore, in our opinion, other mechanisms, besides the one dependent on PIN1-mediated PAT, function to supply auxin for vascular differentiation.

## 4. Materials and Methods

### 4.1. Plant Material and Growth Conditions

*Arabidopsis thaliana* (L.) Heynh. plants were grown at 22 °C with a photoperiod of 10 h light/14 h dark (short day, SD) for 28 days and then transferred to a photoperiod of 16 h light/8 h dark (long day, LD) to induce inflorescence stem development. In order to create the necessary transgenic lines, *Arabidopsis* mutant *pin1-1* ([69]; in En-2 background) was crossed to p*DR5:GFP*, p*YUC1:GUS*, and p*YUC4:GUS* ([44,70]; all in Col-O background), and selected for marker homozygosity.

### 4.2. Histological Analyses

Whole mature inflorescence stems of WT (*n* = 7) and *pin1* mutant plants (*n* = 23) were transversely cut in a series of 30-µm thick sections using a vibratome (Leica VT 1200S; Leica Instruments GmbH, Wetzlar, Germany). The individual stems were cut in an acropetal sequence at intervals of ~0.5 cm. Sections were stained for 1 min in Alcian blue-safranin O or for 5 min in 5% phloroglucinol with 50% HCl [71].

For the auxin response analyses, 1-cm-long apical parts of the inflorescence stems of p*DR5:GFP* (as a control, *n* = 9) and *pin1-p*DR5:GFP (*n* = 17) plants were cut longitudinally or transversely on the series of 25-um thick sections using the vibratome.

To detect the GUS signal in transgenic lines p*YUC1:GUS* (*n* = 30 for WT, *n* = 76 for *pin1* background) and p*YUC4:GUS* (*n* = 30 for WT, *n* = 85 for *pin1* background), 1-cm-long apical parts of the inflorescence stems were incubated at 37 °C for 16 h in a mixture of 1.4 mM XGlcA (Duchefa Biochemie, Haarlem, The Netherlands), 1 mM K_3_Fe(CN)_6_, 1 mM K_4_Fe(CN)_6_, 50 mM sodium phosphate buffer (NaH_2_PO_4_ × Na_2_HPO_4_, pH = 7.0), and 0.1% Triton X-100. Subsequently, the material was fixed in FAA (Formaldehyde 5%–Acetic acid 5%–Alcohol 50%) for 1 h and rinsed in 50% ethanol. 

To visualize the three-dimensional (3D) structure of the xylem strands, inflorescence stems of WT plants (*n* = 15) and *pin1* mutants (*n* = 34) were fixed in FAA, hydrated in a graded ethanol series, incubated in 10% KOH for 2–4 days at 37 °C, rinsed in water, and stained with 0.05 mg/mL propidium iodide (PI; Sigma-Aldrich, Steinheim, Germany) for 1 h. 

### 4.3. Auxin Immunolocalization

Apical parts of 1 cm long of the inflorescences of WT plants (*n* = 6) and *pin1* mutants (*n* = 24) were incubated at 4 °C in 3% *N*-(3-Dimethylaminopropyl)-*N*′-ethylcarbodiimide hydrochloride (EDAC; Sigma-Aldrich) dissolved in 0.1× phosphate buffered saline (PBS) (pH = 7.4; Sigma-Aldrich) with 0.1% Triton X-100 (POCH, Gliwice, Poland) for 4 h. Then, the material was fixed 24 h in FAA, rinsed in 50% ethanol, and hydrated in a graded ethanol series. Next, the cross-sections (30 µm thick) and longitudinal sections (25 µm thick) were prepared using the vibratome. Sections were rinsed sequentially in 25 mM PBS (NaH_2_PO_4_ × Na_2_HPO_4_, pH = 7.0) for 5 min, alkaline phosphatase stabilizing buffer (SB; Sigma-Aldrich) for 30 min, 100% methanol at −20 °C for 10 min, and three times in SB for 10 min. Subsequently, they were incubated with the primary anti-IAA mouse monoclonal antibody (Agdia, Elkhart, IN, USA) dissolved 1:20 in 25 mM PBS for 18 h at 4 °C, rinsed three times for 10 min in 25 mM PBS, incubated in the secondary goat anti-mouse antibody Alexa Fluor 488 (Molecular Probes, Eugene, OR, USA) diluted 1:100 in 25 mM PBS for 4 h, at −24 °C, and then rinsed 3 × 10 min in SB. 

### 4.4. Real-Time Polymerase Chain Reaction (PCR)

Fragments of 0.5 mm length from WT plants and *pin1* mutants at different stages of growth were separately collected for triplicate experiments. Total RNA was extracted using the Plant Total RNA Extraction kit (EurX, Gdańsk, Poland). Reverse transcription was performed using 1 μg of total RNA and a High Capacity cDNA Extraction kit (A&A Biotechnology, Gdynia, Poland) according to the manufacturer’s instructions. Real-time quantitative reverse transcription PCR (qRT-PCR) was performed using Real-Time 2× PCR Master Mix SYBR version B (A&A Biotechnology) on a Light cycler 480 Real-Time system (Roche, 632 Mannheim, Germany). Amplification conditions were as follows: denaturation for 1 min (95 °C), 45 cycles of denaturation for 10 s (95 °C), amplification for 10 s according to the primer-specific temperatures (50–60 °C), elongation for 20 s (72 °C), and cooling for 30 s (40 °C). The specificity of the amplification was verified by melting curve analysis. In all experiments, the *ACTIN2* gene was used as a reference. Primer sequences are shown in Supplementary Table S1. Statistical analyses were performed using the Statistica 13 software (StatSoft; North Melbourne, Victoria, Australia). Data had normal distribution (Shapiro–Wilk test) and the *t*-test was used to verify the significance of differences (from *p* = 0.05).

### 4.5. Microscopy

Histological analyses were performed in an epi-fluorescent microscope (BX-50, Olympus Optical Co., Tokyo, Japan) connected to an Olympus DP71 camera (Olympus Optical Co.) using the Cell^B software (Olympus Optical Co.). The distribution of the GUS signal was analyzed using a stereomicroscope (SMZ745T, Nikon Instruments Europe B.V., Warszawa, Poland) and a digital camera (DLT-Cam PRO, Delta Optical, Nowe Osiny, Poland). The 3D structure of xylem strands was analyzed in a confocal microscope (LSM, FluoView1000, Olympus Optical Co.). Distances from the youngest differentiated xylem elements to the SAM were measured as described Banasiak [34]. The excitation/emission light were 490/590 nm for GFP and AlexaFluor488 and 540–565/632 nm for PI.

## 5. Conclusions

The results presented in this paper answer several important questions related to the mechanisms regulating the formation of the lateral organs on the meristem and the differentiation of the vascular strands associated with them. We showed that the lack of PAT in *pin1* mutants does not fully block these two processes, as other mechanisms that provide auxin remain active. One of these mechanisms could be the transport of auxin to the SAM, with the use of vascular tissues, while another mechanism could be the biosynthesis of auxin directly in the meristem and developing organs. Additionally, we directly showed, for the first time, that auxin is always present in the meristem, independent of PAT. However, auxin presence in the L1 layer is fundamental for organogenesis. In *pin1* mutants, the stable presence of auxin in inner layers of the meristem is ensured by its transport through the vascular tissues. Nevertheless, its presence in the L1 layer probably depends on a change in the auxin transport mode in these tissues and, in older shoots, the induction of an auxin biosynthetic pathway. Deciphering the mechanism(s) changing auxin transport in the vascular tissues, as well as determining whether the mechanisms responsible for organogenesis and vascular differentiation in *pin1* mutants are activated by PAT damage, and whether these mechanisms also interact with PAT in wild-type plants, require further research.

## Supplementary Materials

Supplementary materials can be found at http://www.mdpi.com/1422-0067/20/1/180/s1.
