# Supplementary material for: Ontogenetic Changes in Auxin Biosynthesis and Distribution Determine the Organogenic Activity of the Shoot Apical Meristem in pin1 Mutants
Source: Int J Mol Sci. 2019 Jan 6;20(1):180. doi: 10.3390/ijms20010180 (PMC6337202; doi:10.3390/ijms20010180)
Supplement: Supplementary file 1 [file ijms-20-00180-s001.zip › Supplementary Figure S1.pdf]

Supplementary Figure S1. Secondary growth in the inflorescence stems

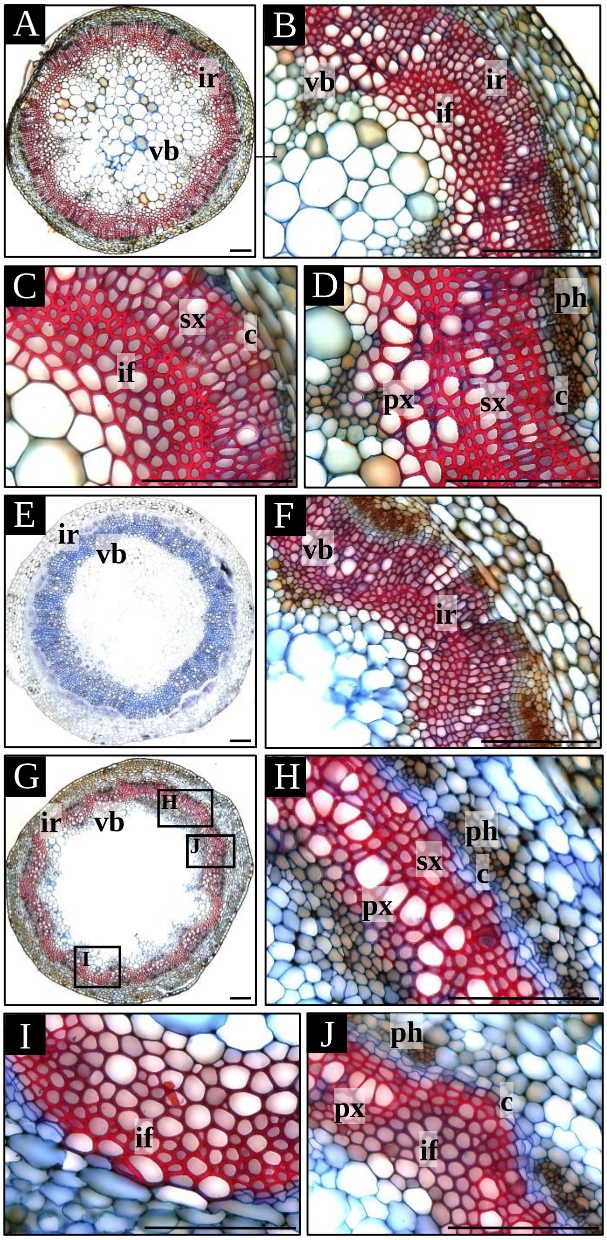

Supplementary Figure S1. Secondary growth in the inflorescence stems. Transversal sections through the basal region of the inflorescence stems of WT (**A-D**) and *pin1* mutant (**E-J**) plants. In WT plants the secondary vascular tissues form a continuous cylinder (**A**). Cambium is present in the interfascicular region (**B,C**) and in the vascular bundles (**D**). In *pin1* mutants the secondary vascular tissues form a continuous cylinder (**E,F**) or develop only locally (**G**) with cambium present in the bundles (**H**) and only sectorially in the interfascicular region (**I,J**). vb - vascular bundle, px - primary xylem, sx - secondary xylem, ph - phloem; if - interfascicular fibers, ir- interfascicular region, c - cambium. Scale bar 100μm.
