# Supplementary material for: Ontogenetic Changes in Auxin Biosynthesis and Distribution Determine the Organogenic Activity of the Shoot Apical Meristem in pin1 Mutants
Source: Int J Mol Sci. 2019 Jan 6;20(1):180. doi: 10.3390/ijms20010180 (PMC6337202; doi:10.3390/ijms20010180)
Supplement: Supplementary file 1 [file ijms-20-00180-s001.zip › Supplementary Figure S2.pdf]

Supplementary Figure S2. Disorders in the collateral structure of the vascular bundles in the *pin1* mutant.

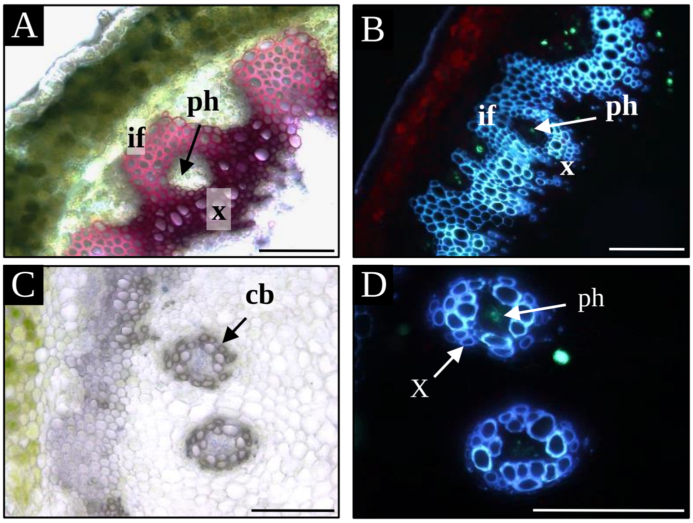

Supplementary Figure S2. Disorders in the collateral structure of the vascular bundles in the *pin1* mutant. Transversal sections through the inflorescence stems stained with floroglucinol (**A**) and alcian blue (**B-D**). The regions with phloem being surrounded by interfascicular fibers and xylem (**A,B**), or only by xylem (**C,D**). x - xylem, ph - phloem, if - interfascicular fibers, cb - concentric bundles. Scale bar 100µm
