# Supplementary material for: Ontogenetic Changes in Auxin Biosynthesis and Distribution Determine the Organogenic Activity of the Shoot Apical Meristem in pin1 Mutants
Source: Int J Mol Sci. 2019 Jan 6;20(1):180. doi: 10.3390/ijms20010180 (PMC6337202; doi:10.3390/ijms20010180)
Supplement: Supplementary file 1 [file ijms-20-00180-s001.zip › Supplementary Table S1.docx]

**Supplementary Table S1. The primers used in qRT-PCR**

| *ACT* | FOR- ATCGAGAAGAACTATGAATTAC; REV- AAGTGCTGTGATTTCTTT |
| --- | --- |
| *YUC1* | FOR- CGGTCGGATTCAATAGCATCTC; REV- AAGCGTAGGACTCAAGGTAGG |
| *YUC4* | FOR- AATGTACCCGATTGGCTTAAGGAG; REV- ACCCACCGTGTATAGTCCCTTC |
| *TAA1* | FOR- TTCGTGGTCAATCTGGATCATGG ; REV- ACCACGTATCGTCACCGTACAC |
