# Supplementary material for: Ontogenetic Changes in Auxin Biosynthesis and Distribution Determine the Organogenic Activity of the Shoot Apical Meristem in pin1 Mutants
Source: Int J Mol Sci. 2019 Jan 6;20(1):180. doi: 10.3390/ijms20010180 (PMC6337202; doi:10.3390/ijms20010180)
Supplement: Supplementary file 1 [file ijms-20-00180-s001.zip › Supplementary Table S2.docx]

**Supplementary Table 2**. Frequencies of *pin1* mutant phenotypes (in %).

| Stem phenotypes | The height of the stems | | | |
| --- | --- | --- | --- | --- |
|  | < 1 cm  n = 43 | 3-6 cm  n = 56 | 15-20 cm  n = 52 | > 20 cm  n = 128 |
| depleted of organs | **93,35** | 12,5 | - | - |
| with single bulges  or organs | 4,65 | **75** | 13,46 | - |
| with multiple bulges, folds and organs | - | 5,36 | **48,08** | - |
| terminated with single flower-like structure | - | - | 3,84 | **24,22** |
| terminated with multiple malformed organs | - | - | 5,77 | **32,81** |
| terminated with meristem necrosis | - | - | 9,62 | **20,31** |
| fasciation | - | 7,14 | 19,23 | **22,66** |
| The table represents the results from one experimental set-up. | | | | |
