# Supplementary material for: Ontogenetic Changes in Auxin Biosynthesis and Distribution Determine the Organogenic Activity of the Shoot Apical Meristem in pin1 Mutants
Source: Int J Mol Sci. 2019 Jan 6;20(1):180. doi: 10.3390/ijms20010180 (PMC6337202; doi:10.3390/ijms20010180)
Supplement: Supplementary file 1 [file ijms-20-00180-s001.zip › Supplementary Table S3.docx]

**Supplementary Table S3**. The distance between the most apical protoxylem element and the SAM.

| Developmental stage | n | Distance range  (μm) | Mean distance  (μm) | sd |
| --- | --- | --- | --- | --- |
| WT stage I-III | 10 | 43-161 | 93.3 | ± 33.04 |
| *pin1* stage I | 7 | 437-1007 | 602 | ± 201.99 |
| *pin1* stage II | 8 | 326 - 441 | 373.75 | ± 44.2 |
| *pin1* stage III | 8 | 139 -221 | 171.25 | ± 27.59 |
| n - number of analyzed stems  sd – standard deviation, related to the mean distance measurements | | | | |
